# Regulation of life span by mitochondrial respiration: the HIF-1 and ROS connection

**DOI:** 10.18632/aging.100292

**Published:** 2011-03-06

**Authors:** Ara B. Hwang, Seung-Jae Lee

**Affiliations:** ^1^ Division of Molecular and Life Science; ^2^ School of Interdisciplinary Bioscience and Bioengineering; ^3^ World Class University Information Technology Convergence Engineering, Pohang University of Science and Technology, Pohang, Kyungbuk, 790-784, South Korea

## Abstract

A mild reduction in mitochondrial respiration extends the life span of many species, including *C. elegans.* We recently showed that hypoxia-inducible factor 1 (HIF-1) is required for the acquisition of a long life span by mutants with reduced respiration in *C. elegans.* We suggested that increased levels of reactive oxygen species (ROS) produced in the respiration mutants increase HIF-1 activity and lead to this longevity. In this research perspective, we discuss our findings and recent advances regarding the roles of ROS and HIF-1 in aging, focusing on the longevity caused by reduced respiration.

For the last two decades, many genes and pathways have been shown to regulate aging across phyla. Among these, impaired mitochondrial respiration extends the life span of yeast, *C. elegans*, *Drosophila*, and mice [1-10]. Although several studies have proposed underlying mechanisms by which diminished mitochondrial respiration promotes longevity, including gene expression changes, global metabolic shift, and changes in energy utilization [11-15], the key genetic factors that mediate this long life span were poorly understood. We proposed that longevity caused by inhibition of mitochondrial respiration in *C. elegans* is promoted by HIF-1 activity via reactive oxygen species (ROS) [16]. Here, we discuss recent findings regarding the roles of HIF-1, ROS, and mitochondrial respiration in the regulation of aging.

## The role of HIF-1 in aging

### HIF-1 in mammalian cellular senescence

HIF-1α (hypoxia-inducible factor 1α) has been identified as the master regulator for cellular adaptation to hypoxia [17]. Under normal oxygen conditions, HIF-1α is maintained in a hydroxylated state by the HIF prolyl hydroxylase (prolyl hydroxylase-domain protein, PHD), leading to degradation of HIF-1, which is mediated by the E3 ubiquitin ligase von Hippel Lindau (VHL). Under low oxygen concentrations, the HIF prolyl hydroxylase does not hydroxylate HIF-1α. Therefore, HIF-1α is stabilized and translocated to the nucleus, where it forms a complex with HIF-1β. This HIF-1 complex binds to HIF-responsive elements (HREs) and turns on various genes to evoke immediate and long-term responses to hypoxia [17, 18]. This HIF-1-mediated transcriptional response has been shown to be crucial for many physiological processes, such as the adaptive response to hypoxia, angiogenesis, vasculogenesis, axon guidance, and aging [17-20].

Since it was first shown in the 1970s that exposing mammalian cells to low oxygen conditions extends their cellular life span [21], extensive research has been performed to understand the role of hypoxia and HIF-1 in cellular aging. *In vivo* microenvironment for hematopoietic stem cells is hypoxic, and stabilized HIF-1α is required to maintain their stem cell-like properties [22]. Mesenchymal stem cells cultured at an oxygen concentration of 3% showed delayed replicative senescence compared with cells cultured in ambient atmospheric conditions of ~20% O_2_ [23]. It has also been shown that aged cells display a decreased ability to express HIF-1 target genes under hypoxic conditions [24] and impaired binding of HIF-1 to HREs [25]. These observations may explain the susceptibility of aged organisms to hypoxic stress [24, 25]. Together these studies suggest that oxygen limitation and/or activation of HIF-1 play important roles in cellular senescence.

### Regulation of the life span of C. elegans by HIF-1

Recent studies using *C. elegans* revealed the role of HIF-1 as a regulator of aging in the animal [16, 19, 26-32]. Initial characterization of *hif-1* mutants showed that *C. elegans* also requires the HIF-1-dependent hypoxic response mechanism to adapt to hypoxic stress [33]. *C. elegans hif-1* mutants are unique among multicellular model organisms because *hif-1* null mutants are viable under normoxia [33]. Because of this viability, it has been straightforward to examine the role of HIF-1 in the regulation of life span.

When *C. elegans* is cultured in low oxygen, its life span is extended [34], raising the possibility that HIF-1 may promote the longevity of the animal. It has been possible to test this idea by asking whether up-regulation of HIF-1 can extend lifespan [16, 19, 26-32]. As in mammals, *C. elegans* EGL-9 (HIF prolyl hydroxylase) and VHL-1 (von Hippel Lindau 1, E3 ubiquitin ligase) are required for the degradation of HIF-1 [35,36]. Several groups have shown that down-regulation of *vhl-1*/E3 ubiquitin ligase or *egl-9*/HIF prolyl hydroxylase as well as overexpression of *hif-1* significantly increases lifespan [16, 27, 29, 30, 32]. However, many issues regarding life span regulation by HIF-1 signaling remain unresolved. For example, the long life span of *vhl-1*/E3 ubiquitin ligase mutants was shown to be completely [30] or partially [32] dependent on *hif-1* but in other reports the longevity caused by *egl-9*/HIF prolyl hydroxylase mutants was shown to be independent of *hif-1*[29]. Remarkably, several groups have reported that *hif-1* deletion also extends lifespan [16, 26-29, 31]. Furthermore, the role of insulin/IGF-1 pathway [16, 26-28, 30] or dietary restriction [16, 28, 30] in the regulation of life span by HIF-1 signaling appear to be very complex [16, 19, 26-30, 32] (see Leiser and Kaeberlein 2010 for an extensive review on these complicated life span phenotypes caused by *hif-1* signaling mutants [19]).

How can we resolve this controversy over the involvement of HIF-1 in signaling pathways modulating the life span of *C. elegans*? We propose that differences in the life span assay may largely account for this discrepancy. For example, one noticeable difference in experimental conditions is the temperature used for the life span measurement. Both Mehta et al. and our laboratory performed life span experiments at 20°C [16, 30], whereas Chen et al. introduced a temperature shift from 20°C to 25°C at the final larval stage prior to measuring adult life span [28]. Temperature is an environmental factor that critically influences life span: for example, worms live for a significantly shorter time at 25°C than at 20°C [37]. We previously showed that mutants with defects in thermosensory neurons have an even shorter life span at 25°C than wild-type animals and have a life span indistinguishable from that of wild type at 20°C [38]. From these data, we proposed that thermosensory neurons moderate the life span-shortening effect of the warm temperature (25°C) [38]. Interestingly, the temperature-dependent life span phenotype of *hif-1* mutants is the opposite of that of the mutants with thermosensory defects: *hif-1* mutants have a long life span when they are cultured at 25°C or shifted from 20°C to 25°C [16, 26, 28] and display no life span phenotypes at low temperatures (20°C and 15°C) [16, 26, 30]. Although this life span shortening is partly due to a temperature-dependent vulval integrity phenotype [26], the temperature-dependent longevity of *hif-1* mutants remains largely intact even after excluding the contribution of the vulval integrity phenotype. Interestingly, HIF-1 has been shown to play a role in heat acclimation in *C. elegans* [39]. Therefore, it will be exciting to test whether this heat acclimation and/or thermosensation is involved in the effects of HIF-1 on the longevity response to temperature.

## Mechanisms of the extension of life span by impaired mitochondrial respiration

### The role of reactive oxygen species

Mild inhibition of the mitochondrial electron transport chain (ETC) increases life span in many species [1-10]. In *C. elegans*, mutations in ETC genes such as *clk-1* (which encodes a mitochondrial hydroxylase involved in ubiquinone production), *isp-1* (which encodes Rieske iron-sulfur protein in complex III), and *nuo-6* (which encodes a subunit of NADH dehydrogenase complex) [1-3, 40] have been shown to extend life span. RNAi knock-down of mitochondrial ETC components also increases the life span of *C. elegans,* and that of *Drosophila* as well [6, 9]. In mice, mutants that exhibit decreased mitochondrial respiration such as *Surf1^−/−^* (mutation in cytochrome c oxidase) [8] and *Mclk1^+/−^* (heterozygous knockout of the mouse homolog of *clk-1*) have long life spans [7]. These findings support the notion that reduced mitochondrial respiration leads to longevity in mammals and suggest the existence of evolutionarily conserved underlying mechanisms.

Because mitochondria are the major source of ROS, one possible mechanism by which a reduction in mitochondrial respiration influences life span may involve changes in the level of ROS. Historically, ROS were believed to be the key causes of aging [41]. The free radical theory of aging proposed that ROS, which are natural byproducts of mitochondrial respiration, cause damage to macromolecules and organelles such as mitochondria. This mitochondrial damage in turn stimulates increased ROS generation, thus evoking a ‘vicious cycle’ that ultimately leads to deterioration of the cell and the organism [41]. However, several studies have shown that many conditions that increase ROS do not decrease life span, in worms and in mice [42, 43, 48]. Moreover, recent studies have highlighted the beneficial role of ROS in longevity [16, 31, 44-48]. For example, antimycin treatment that blocks mitochondria complex III and thus impairs ETC function, triggers excessive ROS production in the mitochondria [49]. Interestingly, antimycin treatment increases the life span of *C. elegans* [5]. Consistent with this, several studies have shown that long-lived *clk-1*, *isp-1,* and *nuo-6* mutant worms have increased ROS levels [16, 31, 50]. Likewise, long-lived *Mclk1^+/−^* mice exhibit decreased respiration rates and elevated H_2_O_2_ levels [7, 51]. These data raise the intriguing possibility that increased ROS may play a causal role in the longevity conferred by the inhibition of mitochondrial respiration.

The Hekimi group recently addressed this issue directly and showed that increased ROS are required for the longevity of *isp-1* and *nuo-6* mutants [31]. They reported that *isp-1* and *nuo-6* mutants have increased superoxide levels, whereas *clk-1* mutants have elevated overall ROS levels. N-acetylcysteine (NAC), a well-defined antioxidant, significantly decreased the life span of *isp-1* and *nuo-6* mutants but had little or no effect on the life span of wild-type animals. In case of *clk-1* mutants, NAC treatment had marginal effects on life span [31]. In addition, three groups independently showed that wild-type worms treated with the ROS-generating chemicals juglone or paraquat are long lived [16, 31, 45]. These studies are consistent with the previous finding by Schulz et al. that restriction of glucose metabolism increases life span through elevating ROS and that antioxidant treatment suppresses this longevity [46]. Together, these data suggest that increased ROS generation in mitochondrial respiration-defective mutants promotes longevity in *C. elegans*.

In contrast to the long-lived mitochondrial respiration mutants described above, a mutant allele of *mev-1*, which encodes a cytochrome b large subunit in complex II [52, 53], cause a short life span [34, 52]. The *mev-1* mutants were initially characterized in an EMS mutagenesis screen to identify mutants that are hypersensitive to paraquat treatment [52], and were subsequently shown that these mutants display increased ROS levels [54]. Studies in our laboratory and by Dingely et al. showed that *mev-1* mutants have even higher ROS levels than the long-lived *clk-1* or *isp-1* mutants [16, 55]. Therefore, it is possible that *mev-1* mutants are short lived because of excessive ROS, which is similar to the conditions that decrease life span by treatment of high concentrations of ROS-generating chemicals [16, 45].

### The role of HIF-1 in longevity induced by impaired respiration and ROS

How do ROS extend the lifespan of respiration mutants? Consistent with experimental results using cultured mammalian cells, recent studies with model organisms show that HIF-1 is stabilized when ROS levels are increased. *Mclk1^+/−^* mice, which display reduced mitochondrial respiration, exhibit elevated ROS levels [51] and increased HIF-1α protein levels under normal oxygen conditions [56]. In addition, the increase in HIF-1 levels caused by RNAi knock-down of *Mclk1* was abolished by a H_2_O_2_-specific antioxidant peptide targeted to mitochondria [56]. In *C. elegans,* we showed that defects in mitochondrial respiration elevated ROS levels, which lead to increased HIF-1 activity [16]. We found that *clk-1* and *isp-1* mutations induced several HIF-1 target genes and that the extended life span promoted by *clk-1* and *isp-1* mutations requires the HIF-1 transcription factor. Moreover, treating the worms with paraquat led to up-regulation of a HIF-1 target gene in a *hif-1*-dependent manner [16]. Together these data suggest that mutations affecting mitochondrial respiration lengthen life span by increasing ROS levels and HIF-1activity. A recent paper reported that an *E. coli* TCA cycle-related mutant that cannot provide reducing equivalents used in the ETC, and thus has reduced respiration, showed an extended chronological life span [57]. Interestingly, the longevity of these mutants required ArcA, which is the functional homolog of eukaryotic HIF [57], suggesting that this longevity regulation is functionally conserved between prokaryotes and eukaryotes.

Several issues regarding the role of HIF-1 in the longevity caused by increased ROS remain to be addressed. First, controversy remains regarding how HIF-1 is activated under low oxygen conditions. One unexpected observation regarding hypoxia is that cells exhibit increased ROS levels under hypoxia [58-60]. Although controversial, several recent studies proposed that mitochondrial complex III is the main source of ROS required for HIF-1 stabilization [61-63]. Under normoxic conditions, growth factors required for vasculogenesis and cytokines required for the maintenance of hematopoietic stem cells stabilize HIF-1, and this stabilization requires ROS [64, 65]. It has been suggested that ROS oxidize Fe^2+^, the coactivator of the HIF prolyl hydroxylase, to Fe^3+^ through Fenton's reaction in cultured mammalian cells [66]. Follow-up on our study showing that increased ROS lead to the activation of HIF-1 in *C. elegans* will be crucial to examine this issue *in vivo*. Second, what are the HIF-1 target genes that mediate the longevity induced by elevated ROS levels? In cultured mammalian cells, increased ROS levels under hypoxia prolong their replicative life span via stabilization of HIF-1, which leads to up-regulation of human telomerase reverse transcriptase (hTERT) and subsequent extension of telomeres [67, 68]. It will be important to determine which HIF-1 target genes are responsible for paraquat-induced longevity in *C. elegans*. Third, because the *hif-1* mutation only partially suppresses the paraquat-induced longevity and because not all *hif-1*-dependent hypoxia genes were induced by inhibiting respiration [16], additional genes appear to be required in parallel to the conventional HIF-1 signaling pathway. Identification of these genes will increase our understanding of the mechanisms by which increased ROS extend life span.

### Mitochondrial unfolded protein response

In contrast to the mitochondrial respiration mutants, we found that longevity caused by RNAi targeting ETC components showed only a partial dependency on *hif-1*[16]. Together with findings that the effect of respiratory-chain RNAi and respiration mutants may exert different outputs in gene expression and metabolism [11, 13], these studies suggest that there are additional mechanisms by which impaired ETC function (in particular that induced by RNAi) increases life span. Recently, several groups showed that inhibition of mitochondrial respiration triggers the mitochondrial unfolded protein response (UPR^mito^) [40, 69, 70] and UPR^mito^ has been shown to be required for the longevity caused by impaired mitochondrial respiration [69]. An especially striking finding of Durieux et al. is that RNAi against an ETC component in a single tissue induced longevity of the whole animal, and this RNAi effect conveys a cell non-autonomous signal to neighboring tissues to elicit UPR^mito^ [69]. Specifically, Durieux et al. showed that RNAi knock-down of the ETC component *cco-1* (cytochrome c oxidase-1 subunit Vb/COX4) in the intestine or neurons is sufficient to extend the life span of *C. elegans*. In addition, they showed that *cco-1* RNAi in neurons causes UPR^mito^ in the intestine and coined the term “mitokine” for the unidentified signaling molecules that presumably relay the signal induced by reduced mitochondrial respiration in one tissue to other tissues [69]. Based on these results, and together with the suggestion that ROS and HIF-1 mediate the longevity conferred by a reduction in mitochondrial respiration, it is tempting to speculate that ROS such as hydrogen peroxide might travel locally and that HIF-1-target genes propagate a signaling cascade in various tissues to promote longevity.

## CONCLUSION

**Figure 1.**
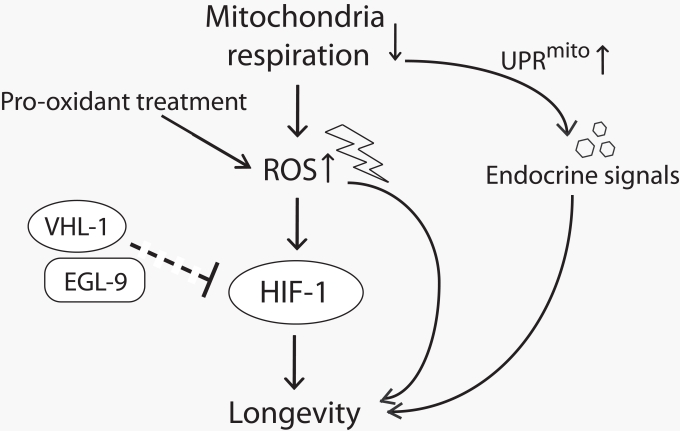
A model for lifespan extension by mild inhibition of mitochondrial respiration in *C. elegans* Mild reduction in mitochondrial respiration leads to the increase in reactive oxygen species (ROS) production and triggers mitochondrial unfolded protein response (UPR^mito^). The increased ROS either by reducing mitochondrial respiration or by pro-oxidant treatment promotes longevity at least in part by increasing the activity of hypoxia-inducible factor 1 (HIF-1). EGL-9 (HIF prolyl hydroxylase) and VHL-1 (von Hippel Lindau 1, E3 ubiquitin ligase) affect longevity perhaps through down-regulating HIF-1 (note that this part is currently inconclusive and therefore is shown with a dashed line.). UPR^mito^ caused by impaired mitochondrial respiration extends lifespan through unidentified endocrine signals that relay the longevity response among different tissues.

In this research perspective, we reviewed recent findings about the roles of HIF-1 and ROS in the regulation of aging, focusing on their roles in life span extension induced by impaired mitochondrial respiration in *C. elegans* (Figure 1). Many interesting questions remain unanswered. Which tissues and functional target genes are important in the regulation of aging by HIF-1? How can both up-regulation and down-regulation of HIF-1 promote longevity? What is the molecular mechanism by which mitochondrial ROS stimulates HIF-1 activity? Future studies using *C. elegans* will be crucial to address these important issues. Since many aging-regulatory processes are conserved between *C. elegans* and mammals, these studies may also provide insights into the regulatory mechanisms of aging in mammals, including humans. Moreover, in addition to aging, HIF-1 and mitochondrial impairment have been implicated in various human diseases such as cancer, diabetes, and neurodegenerative diseases. Thus, we believe that these future studies will help us better understand the pathophysiology of these diseases.
